# Real-Time-Capable Detection of Glottic, Supraglottic and Hypopharyngeal Lesions Using Artificial Intelligence During Flexible Endoscopy

**DOI:** 10.3390/cancers18162609

**Published:** 2026-08-13

**Authors:** Nathalie F. van Rhee, Celine M. L. H. Wilmes, Hidde K. Krijnen, Mischa Hofman, Jonathan Woodburn, Rosanne C. Schoonbeek, Inge Wegner, Henri A. M. Marres, Michel R. M. San Giorgi, Gyorgy B. Halmos, Guido B. van den Broek, Boudewijn E. C. Plaat, David J. Wellenstein

**Affiliations:** 1 Department of Otorhinolaryngology and Head and Neck Surgery, University Medical Center Groningen, Hanzeplein 1, 9713 GZ Groningen, The Netherlands; n.f.van.rhee@umcg.nl (N.F.v.R.); h.k.krijnen@umcg.nl (H.K.K.); m.hofman03@umcg.nl (M.H.); r.c.schoonbeek@umcg.nl (R.C.S.); g.b.halmos@umcg.nl (G.B.H.); b.e.c.plaat@umcg.nl (B.E.C.P.); 2 Department of Otorhinolaryngology and Head and Neck Surgery, Radboud University Medical Center, Geert Grooteplein Zuid 10, 6525 GA Nijmegen, The Netherlands; celine.wilmes@radboudumc.nl (C.M.L.H.W.); henri.marres@radboudumc.nl (H.A.M.M.); guido.vandenbroek@radboudumc.nl (G.B.v.d.B.); 3 WSK Medical B.V., Science Park 301, 1098 XH Amsterdam, The Netherlands; jonathan.woodburn@wskmedical.ai; 4 Department of Otorhinolaryngology, Martini Hospital, Van Swietenplein 1, 9728 NT Groningen, The Netherlands; m.sangiorgi@mzh.nl; 5 Department of Otorhinolaryngology and Head and Neck Surgery, Rijnstate Hospital, Wagnerlaan 55, 6815 AD Arnhem, The Netherlands; david.wellenstein@radboudumc.nl

**Keywords:** artificial intelligence, deep learning, head and neck cancer, supraglottic larynx, hypopharynx, endoscopy

## Abstract

**Background/Objectives**: Supraglottic and hypopharyngeal carcinomas are aggressive malignancies that are often diagnosed at advanced stages. Timely recognition of these malignancies is influenced by many factors, such as endoscope quality and experience. This study evaluated the potential of artificial intelligence (AI) to support real-time detection and classification of such lesions during flexible endoscopy in the outpatient clinic. **Methods**: A previously developed deep learning (DL) algorithm was extended from a glottic lesion model to the unified localization and classification of glottic, supraglottic, and hypopharyngeal lesions during flexible endoscopy. Lesion frames were extracted from endoscopy videos obtained at two head and neck oncology centers and one secondary referral center between 2012 and 2024. These frames were annotated and labeled based on histopathological or clinically confirmed reference diagnoses. The primary outcome was the unified model’s performance in detection of lesions per frame. After training (70% of data), the positive predictive value (precision) and sensitivity (recall) of this model were calculated on an independent test set (30% of data), stratified by subsite and tumor (T-) classification. Secondly, the model’s binary classification performance (benign or malignant) was evaluated. **Results**: From 490 supraglottic and hypopharyngeal endoscopy videos, 40,059 frames with a benign or malignant lesion were extracted and added to the 56,036 glottic lesion frames in the database, comprising 1336 lesion videos and 123 healthy control videos (total *n* = 1459). On the test set, the model achieved a detection precision of 92.1% (95% CI: 90.9–93.2) and a recall of 73.3% (95% CI: 69.8–76.6). Detection performance increased with higher T-stage. Among correctly detected lesion instances in a malignancy-dominated test set (65.3% of lesion instances), sensitivity for malignancy detection was 94.3% (95% CI: 91.8–96.5). Combined end-to-end performance for correct malignant lesion detection and classification was estimated at 69.1% (object-level recall 73.3% × classification sensitivity 94.3%). Evaluation of 33 lesion-free videos demonstrated a mean frame-level specificity of 46.6% and a median of 19 false positive detections per video. **Conclusions**: This is the first study to report a DL model for real-time endoscopic detection and classification of benign and malignant laryngeal and pharyngeal lesions. The developed model showed promising lesion detection and cancer classification performance in the evaluated test set. T1 tumor detection remains an important limitation, particularly because early-stage detection is a primary aim of AI-assisted endoscopy. Further model testing is required in real-world lesion prevalence settings, where external validation and clinical usability should be investigated.

## Simple Summary

Supraglottic laryngeal and hypopharyngeal cancers are notorious for late detection and diagnosis at advanced stages, negatively influencing treatment outcomes and prognosis. Multiple factors contribute to delayed diagnosis, including patient-related and referral-related issues. Early lesions in these anatomically and functionally complex anatomical subsites are easily missed during routine flexible endoscopy, even by experienced clinicians. This study evaluated the potential of a deep learning model to support real-time detection and classification of such lesions during endoscopy of the larynx and hypopharynx. Building upon a previously developed artificial intelligence (AI) model for glottic laryngeal lesions, the algorithm was expanded to a unified database, adding supraglottic and hypopharyngeal lesions. The results of this unified model demonstrate a high positive predictive value (precision) (92.1%) and a promising detection sensitivity (recall) (73.3%). Among correctly detected lesions, the model accurately classified cancers in 94.3% of instances. Combined end-to-end performance for correct malignant lesion detection and classification was estimated at 69.1% (object-level recall 73.3% × classification sensitivity 94.3%). These preliminary results demonstrate the feasibility of a real-time model for detection and classification of laryngeal or hypopharyngeal lesions and support further real-world evaluation. If proven reliable and clinically validated, such a model may reduce the risk of inadequate diagnosis, thereby facilitating earlier detection and potentially improving oncological and functional outcomes.

## 1. Introduction

Head and neck cancer (HNC) predominantly consists of squamous cell carcinomas (SCCs) originating in the anatomically and functionally complex upper aerodigestive tract (UADT) [1]. Among HNC subsites, hypopharyngeal carcinoma is associated with the poorest prognosis, with five-year survival rates declining from approximately 50% in T1-T2 disease to about 25% in T3-T4 disease [2,3,4,5,6]. This poor outcome is primarily attributed to delayed diagnosis, as early symptoms such as globus sensation and referred otalgia are nonspecific. Furthermore, submucosal extension or lymphatic spread may occur even in early-stage disease [7,8]. Supraglottic carcinoma presents similar diagnostic challenges and is also frequently identified at a more advanced stage [9]. Early detection is therefore essential, as treatment of early-stage disease is typically less extensive and associated with improved local control, lower recurrence rates, and better preservation of breathing, swallowing, and speech function. In contrast, advanced-stage disease often requires extensive surgery and/or (chemo)radiotherapy, resulting in substantial morbidity and reduced quality of life [8,10].

Flexible endoscopy is the standard of care for the initial laryngopharyngeal assessment in the outpatient clinic. However, early lesions may be missed during endoscopy due to the anatomical complexity of these regions and the subtle, nonspecific appearance of early mucosal abnormalities [11,12]. Technological advancements, such as high-definition (HD) white-light imaging (WLI) and narrow-band imaging (NBI) have improved mucosal visualization. Nevertheless, diagnostic performance remains highly dependent on clinical experience [12,13,14,15]. Artificial intelligence (AI), specifically deep learning (DL)-based computer-aided detection and diagnosis, has emerged as a promising strategy to improve endoscopic recognition in the UADT, achieving performance levels comparable to expert clinicians [16,17].

Despite advances in AI for laryngopharyngeal endoscopy, most algorithms have been developed and validated for a single anatomical subsite, which restricts their applicability in routine clinical practice [16]. Additionally, most studies have used still images rather than video data, thereby limiting the potential for real-time endoscopic assessment [18,19,20,21,22]. Until recently, AI systems for laryngopharyngeal endoscopy were primarily confined to research settings and were not commercially available for routine clinical use. As a result, evidence supporting real-time AI-assisted diagnosis across multiple UADT subsites remains limited.

Although AI-assisted endoscopy has advanced recently, the real-time performance of DL algorithms for detecting and classifying lesions across multiple laryngopharyngeal subsites remains unestablished. To address this gap, the glottic DL algorithm developed by Wellenstein et al. was expanded into a unified laryngopharyngeal model capable of analyzing lesions in the glottic, supraglottic, and hypopharyngeal regions [23]. This study aimed to develop and internally evaluate this model for lesion localization and classification across the glottic, supraglottic, and hypopharyngeal regions, including assessment of performance across subsites and tumor classification stages. In doing so, we aim to establish a basis for the further development and clinical evaluation of AI-assisted endoscopy.

## 2. Materials and Methods

### 2.1. Study Design

This multicenter diagnostic accuracy study was conducted at the Radboud University Medical Center (Radboudumc) and University Medical Center Groningen (UMCG) in The Netherlands. The feasibility and accuracy of the Zeno AI DL algorithm for localizing and classifying glottic, supraglottic, and hypopharyngeal lesions during flexible endoscopy was evaluated. Originally trained on glottic lesions only, the model was expanded using a combined dataset including lesions from the three subsites [23].

Informed consent was obtained from the patients in accordance with the requirements and approval of the local institutional review boards. Diagnostic performance was reported based on the Standards for Reporting Diagnostic Accuracy using Artificial Intelligence (STARD-AI) 2025 statement and Quality Assessment of Preclinical AI Studies in Diagnostic Endoscopy (QUAIDE) guideline [24,25]. The study received approval from the medical ethics committees of both participating centers (Radboudumc file: 2025-18723; UMCG file: M23.325004). The DL algorithm was developed in collaboration with WSK Medical.

### 2.2. Data Acquisition

The DL algorithm was trained using the previously described “Zeno AI” dataset developed by Wellenstein et al., which included glottic lesions from laryngoscopic videos collected at Radboudumc [23]. The algorithm was subsequently updated with an additional dataset of glottic, supraglottic and hypopharyngeal endoscopy videos collected from two head and neck oncology centers: Radboudumc (between 2012 and 2024) and UMCG (between 2015 and 2024). Additionally, the Martini Hospital in Groningen (MH), a secondary referral center, contributed further glottic cases (dating from 2023 to 2024). Endoscopic videos were downloaded from the electronic patient files in MP4 format. Videos were cut into one in ten frames and stored using the Computer Vision Annotation Tool (CVAT) (Version 2.10.0) [26]. At Radboudumc, transnasal WLI videos were acquired using Pentax transnasal flexible endoscopes (VNL1570STK, VNL9-CP, VNL11-J10, and VNL15-J10; PENTAX Nederland B.V., Dodewaard, The Netherlands). At UMCG, the CV-170 HD Olympus video processor was used (Olympus Medical Systems, Tokyo, Japan). At MH, Olympus Medical Systems (ENF VH connected to CII-S1) were used. In general, video quality was not used as an exclusion criterion to better reflect routine clinical conditions. However, videos or video frames exhibiting permanent motion artifacts or visual obstructions that prevented lesion annotation, such as saliva or blurring, were excluded, as their low quality was not expected to contribute to the development and validation process. Patients who visited the participating centers and underwent an endoscopy without any lesions visible were included as healthy controls. Patients could not be recognized in the videos in any way.

Malignant lesions were defined as SCC and high-grade dysplasia, including carcinoma in situ, confirmed by histopathology according to the most recent WHO classification for head and neck lesions [27]. In line with this classification, low-grade dysplasia was categorized as a benign lesion, while high-grade dysplasia and carcinoma in situ were classified as (pre)malignant. This binary classification was consistently applied throughout the study and may have affected the distribution of lesions between benign and malignant categories. Benign lesions were confirmed either by histopathology or by clinical follow-up of at least three months demonstrating a stable clinical course without evidence of malignant progression. While a three-month follow-up does not entirely exclude the possibility of delayed malignant progression, this approach was considered acceptable for lesions with a benign clinical appearance in routine practice. If there was persistent doubt on the nature of the lesion, these videos were excluded from the database. No biopsies were performed on healthy controls. The database included three classification categories:

• Malignant: SCC and high-grade dysplasia;
• Benign: polyp, cyst, nodule, granuloma, Reinke’s edema, hyperkeratosis, papilloma, hyperplasia, and low-grade dysplasia;
• Normal: videos of healthy controls without laryngeal or pharyngeal lesions.

### 2.3. Data Labeling and Annotation

A single patient video could contain multiple lesions, either benign or malignant, and each video comprised many frames. A lesion visible in a given frame was considered a lesion instance. Thus, a single frame could contain multiple lesion instances, while one video could contain hundreds of lesion instances across its frames. All lesion instances were provided with a ground-truth annotation box at the frame level. Annotations were generated in the You Only Look Once (YOLO) Bounding Box format by four medical doctors trained in endoscopic video evaluation (N.F.v.R., C.M.L.H.W., H.K.K., M.H.) [28]. Lesions were classified according to the reference standard and subsequently converted to a binary label (benign or malignant). Ambiguous cases were resolved through consensus discussions with experienced head and neck surgeons (B.E.C.P., D.J.W., G.B.H., I.W.). Healthy control videos were included without bounding box annotations. In addition, frames without visible lesions were extracted from videos containing lesions and were likewise included without bounding box annotations. The dataset was split at the patient level into a training/validation set (70%) and an independent test set (30%) to ensure that data from the same patient were not present in both sets. The resulting test set contained 34.7% of all lesion frames and 50.5% of all non-lesion frames. These proportions reflect the distribution of frames within the included videos rather than a frame-level sampling strategy, as the number of frames per video and the presence of lesions varied between patients. Within the training/validation set, five-fold cross-validation was performed at the patient level to train the model and optimize hyperparameters. For supraglottic and hypopharyngeal videos, tumor (T)-classification was additionally used as a stratification variable to maintain a comparable stage distribution across the training/validation and test sets. Tumor sublocation and stage were assessed manually based on electronic health records. For cases involving multiple sublocations, tumors were categorized by tumor bulk or weight as observed during endoscopy and as reported in radiology reports. Since this report builds upon previous work, tumor stage and model performance were only available and assessed for supraglottic and hypopharyngeal carcinoma.

### 2.4. Model Development and Training

The model makes use of a two-stage pipeline. First, lesion instances were detected using an algorithm based on the YOLOv8 architecture. Predicted bounding boxes with a confidence threshold (objectness score) of 0.36 or above were regarded as a detection. After detection, the EfficientNet B0 convolutional neural network backbone was used for classification of the lesion. Discrimination between benign or malignant disease can be evaluated on a linear class probability scale ranging from 0 (certain benign) to 1 (certain malignant). A classification probability below 0.341 was determined to be a confident benign classification, and a score of 0.743 and above was a malignant classification. Low confidence classifications ranging between a class probability score between 0.341 and 0.743 were flagged as uncertain. The objectness score and class probability thresholds were determined based on the validation dataset. An elaborate technical description of the model can be found in the Supplementary Materials (doi: 10.5281/zenodo.20996838) [29,30,31,32]. Calibration plots were provided in eFigure 1.

### 2.5. Outcomes and Statistical Analysis

Model performance was evaluated once on the independent held-out test set, and all reported performance metrics are based on this test set. The primary outcome was detection performance, quantified by precision (positive predictive value), recall (sensitivity) and the area under the precision-recall curve (AUPRC), as defined in Table 1. The results are presented on an object-per-frame-level, meaning that for each video-frame, the ground-truth bounding box(es) surrounding the lesion instance (if present) were compared with the prediction of the model. A true positive (TP) object detection was defined by an intersection-over-union (IoU) of ≥0.3 (Table 1). Since looser detections were considered equally important to precise ones, the IoU threshold was set at 0.3. Additionally, a secondary analysis was performed by recalculating the AUPRC using an IoU threshold of 0.5, thereby providing transparency on the effect of a less strict IoU threshold. True negative (TN) rates were not reported at the object-level because each image contains numerous lesion-free regions, making this an uninformative metric. The F1 score was reported as the harmonic mean of precision and recall. Detection metrics were additionally stratified by subsite and T-classification. The DL algorithm was expected to produce no detections for healthy control cases. In addition to object-level detection results, descriptive video-level results were presented. For videos containing at least one lesion, the median number of TPs per video, along with interquartile range (IQR) and range were given. Video-level sensitivity was calculated for each video using all TP detections and dividing them by the total of TP and false negative (FN) detections. Mean sensitivity across all lesion videos and per subsite was calculated by summing individual video sensitivities and dividing them by the total number of videos. Mean and median sensitivity, IQR and range were then reported. For videos without lesions, the median, IQR and range of false positives (FP) were provided. To calculate a specificity for these videos, each frame was regarded as either aTN frame (without any FP detections) or a FP frame (with at least one FP detection). Frames were then aggregated to a video-level specificity by dividing all TN frames by the total of TN and FP frames. The mean specificity across all healthy videos was then determined. Mean specificity was reported alongside the median, IQR and range.

In addition to lesion localization, the DL algorithm was assessed for its capacity to distinguish between benign and malignant lesions. Classification performance was only reported on correctly detected lesion instances. Secondary outcomes included binary classification performance (benign versus malignant), assessed by the sensitivity, specificity, area under the receiver operating characteristic curve (AUROC), accuracy, balanced accuracy and coverage (Table 1). Stratification was performed per subsite. To assess the performance of the model for detecting and correctly classifying (pre)malignant lesions, the detection recall was multiplied by the classification sensitivity, resulting in a combined end-to-end performance metric. To showcase the potential effect of class imbalance in an expected malignancy-dominated prevalence setting, classification analyses on the unified test set were repeated with the fictive malignancy prevalences of 33%, 10% and 2%. These estimates were chosen based on clinical judgment and were intended to represent plausible scenarios rather than established prevalence estimates. 

Inference speed was also assessed to determine real-time applicability. This was measured in frames per second. To be able to provide a 95% confidence interval (CI) for the diagnostic object-level metrics, outcomes were bootstrapped with 2000 iterations. Resampling was performed at the patient level to account for the clustered nature of the data. All analyses were performed using R Statistical Software (v4.4.1; R Core Team 2024).

## 3. Results

### 3.1. Dataset Characteristics

**Figure 1:**
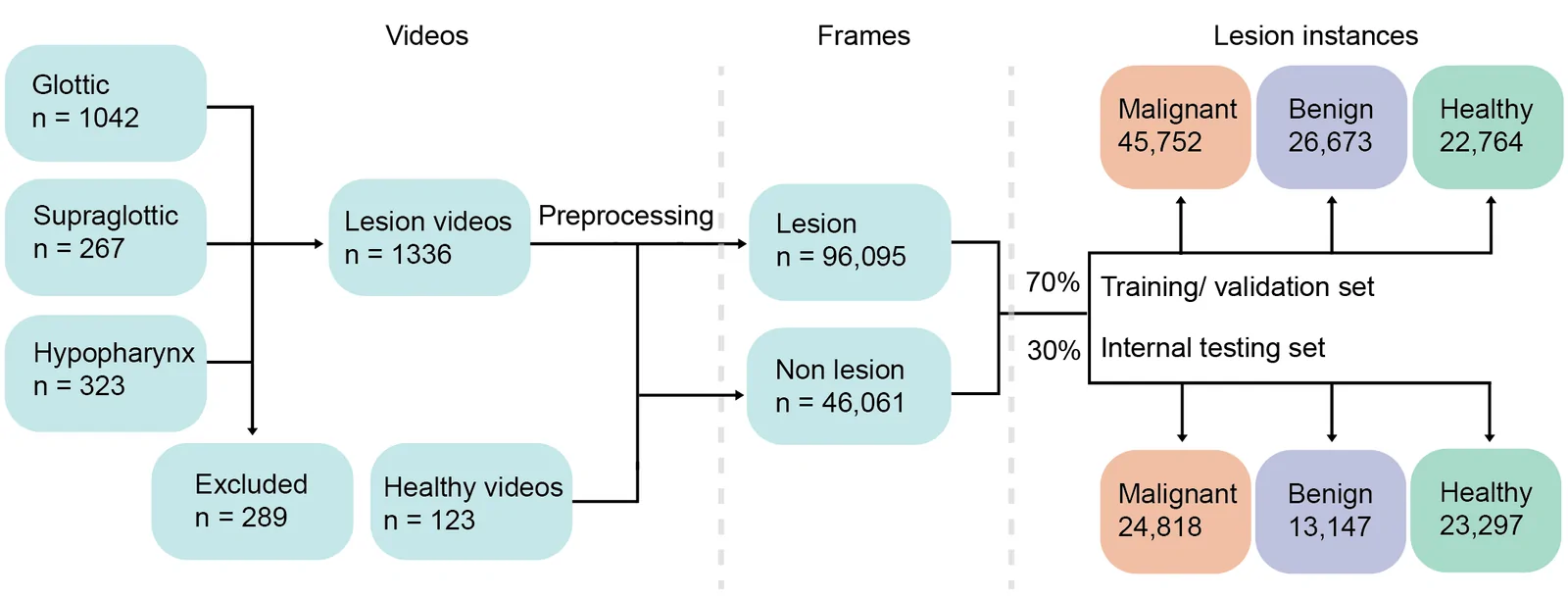
Data acquisition flowchart illustrating the dataset composition, including distribution across subsites, preprocessing steps such as dividing videos into frames, partitioning into training/validation (70%) and internal test (30%) sets and lesion classification categories. Note that one lesion frame may have contained multiple (malignant or benign) lesion instances. One healthy instance is a video frame without a lesion.

A total of 1632 endoscopy videos containing lesions (1042 glottic, 267 supraglottic, and 323 hypopharyngeal) and 123 healthy control videos were collected. In total, 296 videos were excluded due to persistent motion artifacts or obstructions that hindered accurate annotation, resulting in a final dataset of 1336 lesion videos. Following preprocessing (including deletion of video frames meeting exclusion criteria), the resulting database consisted of 142,156 frames, comprising 96,095 lesion frames and 46,061 non-lesion frames. The dataset composition is presented in Figure 1. Carcinoma accounted for 63.9% of all lesion instances in the full dataset and 65.3% in the test set. Among benign lesions, papilloma and polyps were most frequently observed. Table A1 and Table A2 provide an overview of the distribution and frequencies of the lesion types and instances included in the database.

### 3.2. Detection Performance of the DL Algorithm

**Figure 2:**
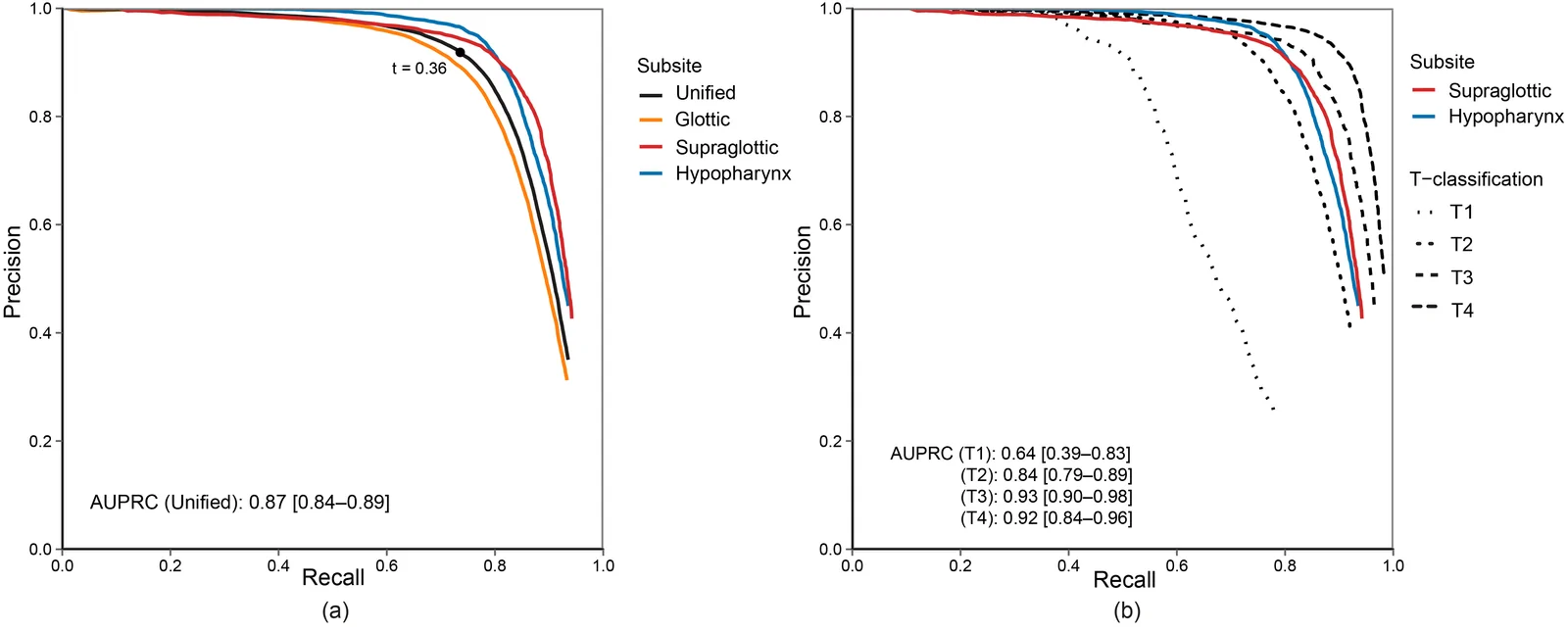
Precision-recall curves for lesion detection by the DL algorithm, at the object level. (**a**) Detection performance stratified by subsite, with the optimal objectness score (t = 0.36) indicated. (**b**) Detection performance stratified by T-classification. Abbreviations: AUPRC, Area under the precision-recall curve.

Across all subsites, the DL algorithm achieved a unified precision of 0.921 (95% CI: 0.909–0.932), a recall of 0.733 (95% CI: 0.698–0.766), an AUPRC of 0.868 (95% CI: 0.836–0.889), and an F1 score of 0.816. The overall results and subsite-stratified breakdown are summarized in Table 2 and depicted in Figure 2a.

Subsite-stratified analysis showed a precision of 0.905 (95% CI: 0.888–0.920) for glottic lesions, 0.932 (95% CI: 0.906–0.953) for supraglottic lesions, and 0.961 (95% CI: 0.948–0.973) for hypopharyngeal lesions. Recall ranged between 0.718 on the glottic subsite to 0.771 on supraglottic instances (Table 2).

To evaluate the clinical relevance of detection performance across disease stages, results were stratified by T-classification (Table 2 and Figure 2b). T1 tumors exhibited the lowest detection performance, with a precision of 0.926 (95% CI: 0.828–0.967), a recall of 0.491 (95% CI: 0.276–0.700), and an AUPRC of 0.640 (95% CI: 0.388–0.834). In contrast, T3 tumors demonstrated the highest performance, with a precision of 0.954 (95% CI: 0.934–0.969), a recall of 0.851 (95% CI: 0.799–0.899), and an AUPRC of 0.932 (95% CI: 0.901–0.958).

**Figure A1:**
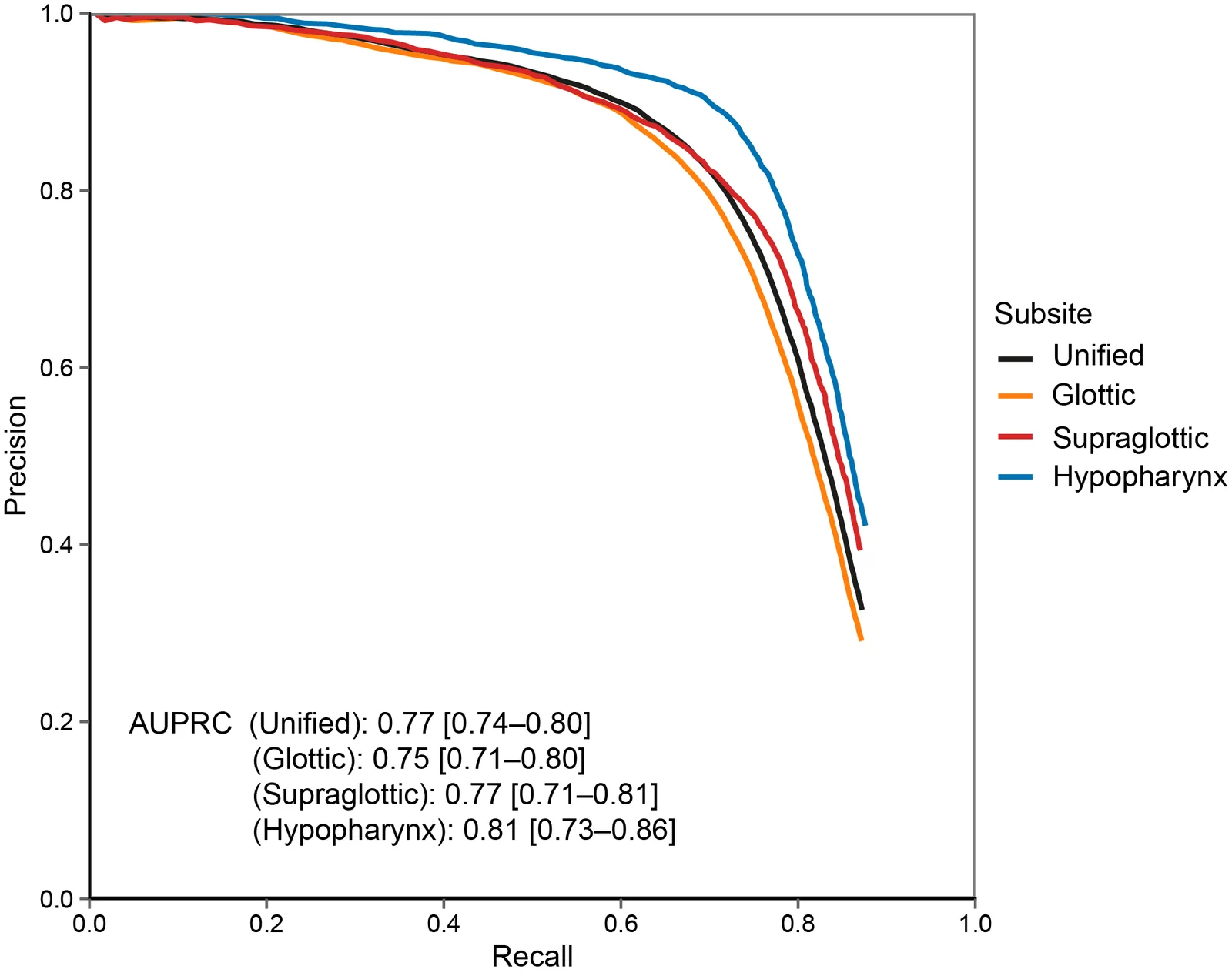
Precision–recall curves and corresponding AUPRC values with 95% confidence intervals for lesion detection at an IoU threshold of 0.5. Although precision remained high, recall was lower than at an IoU threshold of 0.3, as expected with the stricter criterion for a correct detection. Nevertheless, AUPRC values remained high, exceeding 0.75 for all subsites. Abbreviations: AUPRC, Area under the precision-recall curve; IoU, intersection-over-union.

Results of the secondary analysis using an IoU of 0.5 are presented in Figure A1 and show slightly lower AUPRC values between 0.754 and 0.808.

On a video-level, a median of 58 TP detections were found per video (IQR 34–110, range 0–623). There were seven videos (1.8%) without any TP detections: three videos with lesions in the glottis and four videos with lesions in the hypopharynx. Mean overall sensitivity was 0.738, with a median of 0.840 and IQR of 0.586–0.953 (range 0.00–1.00). For the glottis, mean and median sensitivity were 0.749 and 0.843 respectively (IQR 0.585–0.955). Sensitivity on the supraglottic videos was 0.749, and showed a median of 0.833 (IQR 0.628–0.957). Mean hypopharyngeal sensitivity was 0.701 (median 0.819, IQR 0.585–0.950). Of the 33 healthy videos included in the test set, a median of 19 FPs were present per video (IQR 11–52, range 0–212). There were three videos without any FP predictions. Using the frame-level definitions for TN and FPs, the mean specificity was 0.466, with a median of 0.449 and IQR of 0.200–0.714 (range 0.063–1.00).

### 3.3. Classification Performance of the DL Algorithm

**Figure 3:**
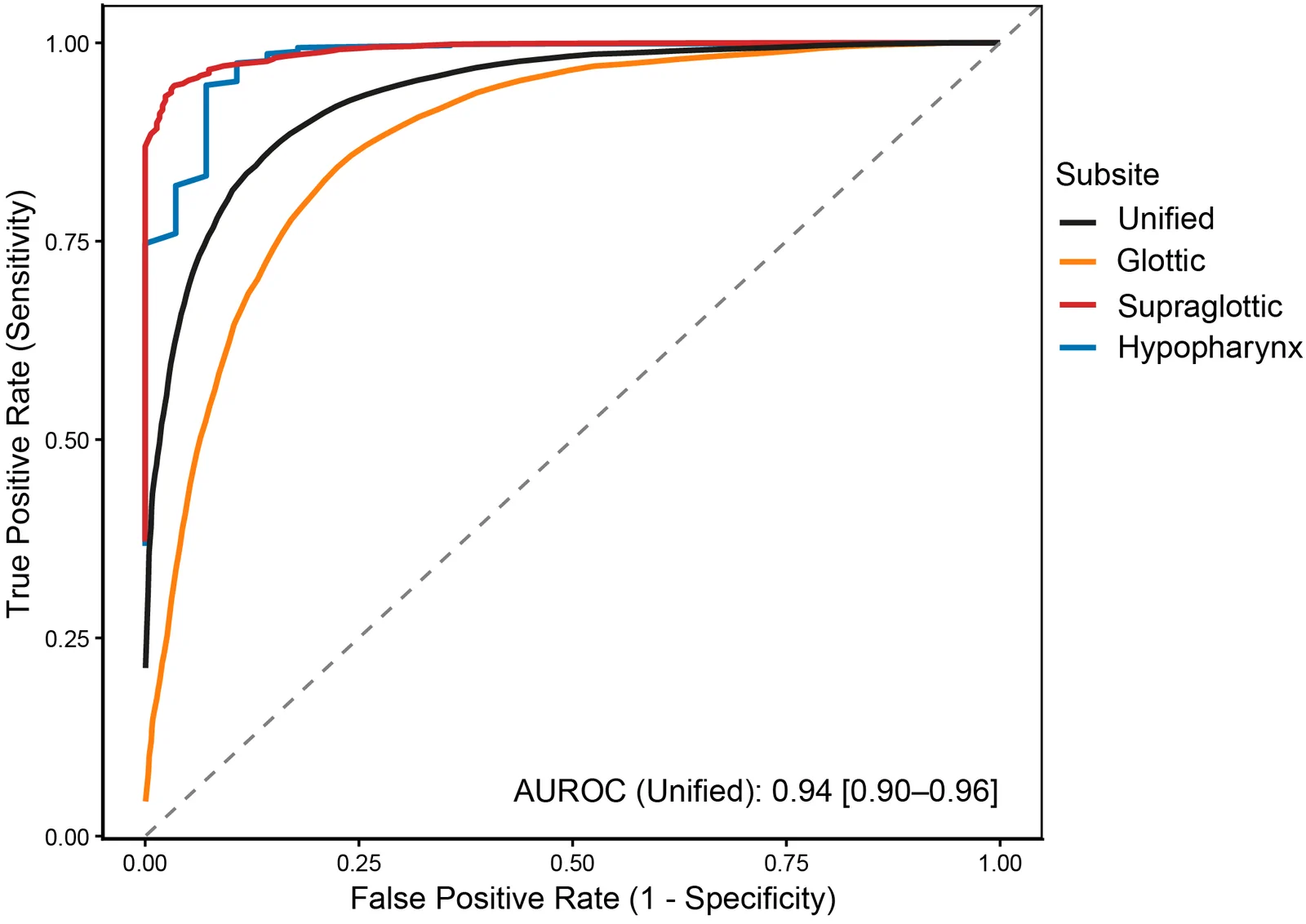
Receiver operating characteristic curves for binary classification by the DL algorithm, stratified by subsite and at the object level. Abbreviations: AUROC, area under the receiver operating characteristic curve.

**Figure 4:**
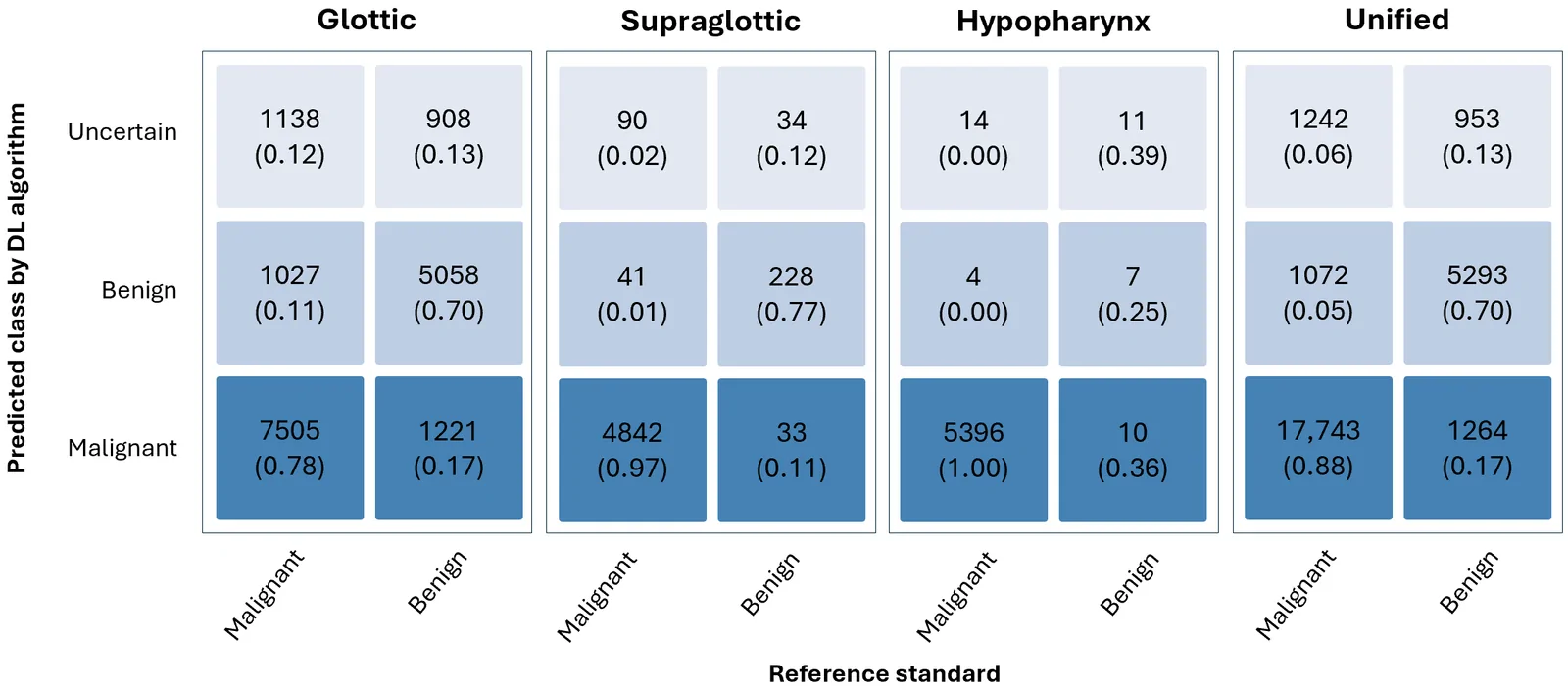
Confidence matrices for binary classifications by the DL algorithm, at the object level. Stratified by subsite.

The model achieved an overall sensitivity for identifying (pre)malignant lesions of 0.943 (95% CI: 0.918–0.965) and a specificity of 0.807 (95% CI: 0.716–0.887). The AUROC was 0.935 (95% CI: 0.899–0.962). The coverage was 0.920 (95% CI: 0.906–0.934), indicating that 8% of class predictions received an uncertain classification. In total, 1242 malignant lesions (6.2%) and 953 benign lesions (12.7%) were censored and classified as uncertain. Binary classification performance is summarized in Table 3 and Figure 3 and Figure 4. Combined end-to-end performance for correct malignant lesion detection and classification was 69.1%.

Classification performance differed by subsite. Sensitivity was highest in the supraglottic and hypopharyngeal subsite. However, the high sensitivity observed in the hypopharynx primarily reflects the highly imbalanced class distribution, with 99.5% of included instances being malignant, rather than superior discriminative performance of the model. Specificity was lowest in the hypopharynx, which was also reflected in a balanced accuracy of 0.706 (Table 3). With constant sensitivity and specificity at 94.3% and 80.7%, the positive predictive value decreased from 90.2% at the observed study prevalence to 70.7%, 35.2%, and 9.1% at malignancy prevalences of 33%, 10%, and 2%, respectively (Table A3). The negative predictive value increased from 88.3% to 96.6%, 99.2%, and 99.9%, respectively.

### 3.4. Inference Time and Real-Time Feasibility

**Figure 5:**
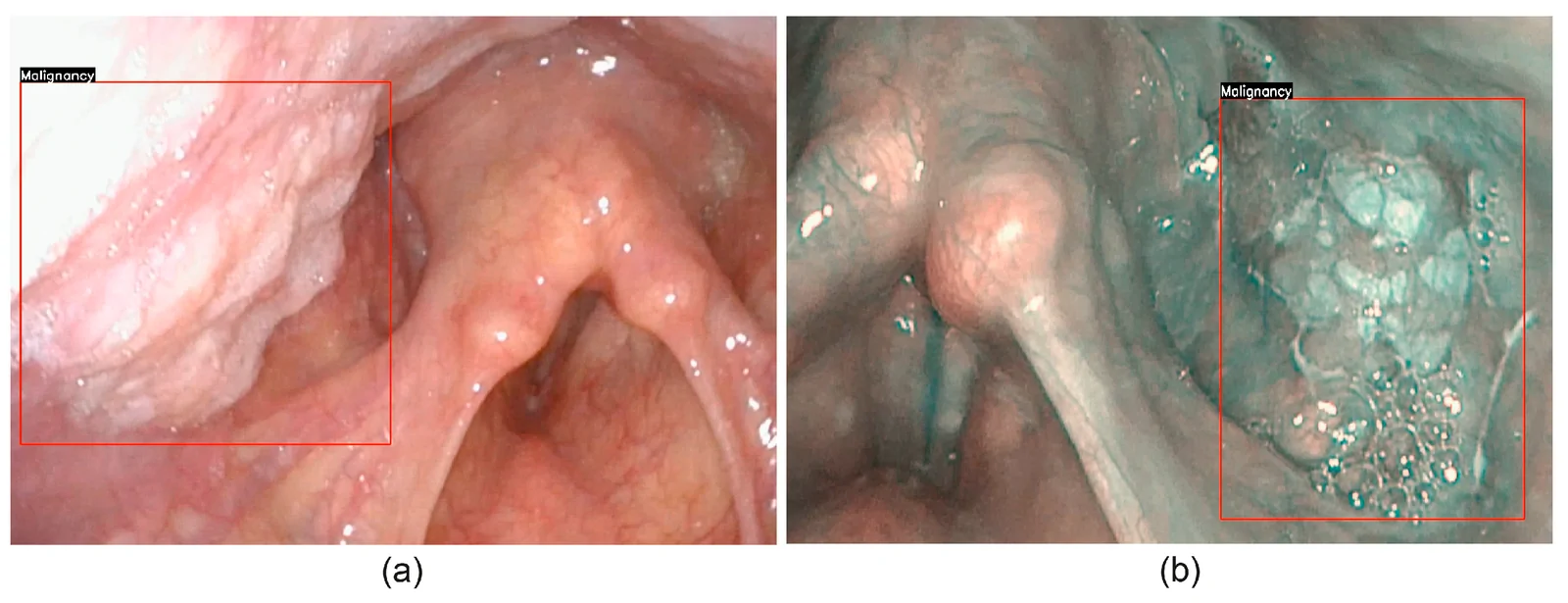
Representative test set video frames illustrating localization and classification of the DL model (full videos available in Supplementary Materials). (**a**) White-light frame of a T1 hypopharyngeal carcinoma in the right pyriform sinus. (**b**) Video-frame with narrow-band imaging of a T1 hypopharyngeal carcinoma in the left pyriform sinus.

Inference speed was evaluated to assess whether the DL algorithm satisfies technical requirements for real-time clinical applications. The full model functions at an inference speed of 76 frames per second on a NVIDIA Medical AI Box-PC ONYX ACCEL-JS1000 (Onyx Healthcare Inc., New Taipei City, Taiwan). Figure 5 represents examples of lesion localization and classification by the DL algorithm on test set frames of T1-classification carcinoma. Figure 5a (Supplementary Video S1) is a representative example of a video with a low recall. Figure 5b, an NBI frame, (Supplementary Video S2) represents a stronger detection performance. It should be noted that the analyses were performed on shortened video versions, from which some frames may have been removed. The full videos are available in the Supplementary Materials section (doi: 10.5281/zenodo.20996838).

## 4. Discussion

This study evaluated a DL algorithm for real-time detection and classification of laryngopharyngeal lesions, extending previous applications on glottic lesions to the supraglottic larynx and hypopharynx. This unified DL model achieved 92.1% precision and 73.3% recall across all subsites. In the evaluated test set, in which 65.3% of lesion instances were malignant, sensitivity for malignancy classification was 94.3%. These results indicate the technical feasibility of a unified model for detecting and classifying laryngopharyngeal lesions in endoscopy videos. However, performance varied across analyses and should be interpreted in the context of the data structure and underlying malignancy prevalence. The results are promising but represent a preliminary model evaluation rather than evidence of clinical diagnostic performance, and further validation in external datasets and prospective clinical settings is needed.

The high object-level detection precision is an important finding, as it may help ensure clinician trust in the system and facilitate its successful integration into clinical practice. Performance on the 33 videos without any lesions showed a median of 19 FP detections per video. Three videos were included without any predictions, whereas there were a few videos in which higher FP rates were found (range 0–212). Overall specificity was just over 40%. However, this video-level analysis does not fully account for the fact that the majority of each frame consists of correctly classified negative areas, which could not be adequately considered. The worst-performing videos were those containing saliva and anatomical variations in nasopharynx and oropharynx.

Tumor-classification stratified analysis demonstrated reduced performance in early-stage disease, reflecting the clinical challenge of detecting subtle lesions in the supraglottic larynx and hypopharynx. Early-stage carcinomas in these regions often exhibit subtle mucosal irregularity, submucosal spread, or limited color contrast with surrounding tissue, featuring difficulties in the detection even for experienced endoscopists [7,8,11]. Kumai et al. found that early hypopharyngeal cancers (Tcis-T2) are frequently missed during routine flexible endoscopy of the UADT, with otorhinolaryngologists reporting a detection rate of only 20% [11]. At first glance, an overall recall of 73.3% in our study may suggest that approximately one in four lesions was entirely missed. However, the reported performance metrics should be interpreted in light of the per-object, per-frame analysis. Each flexible endoscopic examination comprises hundreds to thousands of sequential frames, all evaluated independently by the algorithm. In clinical practice, physicians synthesize information from the entire video sequence rather than relying on individual frames. Consequently, object-level analyses may underestimate real-world performance because a lesion missed in one frame is often detected in subsequent frames. In this study, the model correctly detected an average of 73.3% of lesion instances in the evaluated test set, based on object-level recall analysis. At the video-level, we found that in almost all videos (98.2%), at least one TP was present (median of 58 TPs per video). In seven videos no lesions were picked up by the model. Mean sensitivities on all subsites were higher than 74%. These results provide a more clinically interpretable measure of whether the model detected a lesion somewhere in a video, but still do not fully reflect the complexity of the data and are therefore not an exact measure of lesion-level detection performance. Despite these limitations, the object and video-level results indicate a promising detection capability. Furthermore, it can be argued that, if at least one lesion is found in a video, this can be reason to further analyze a patient and perform additional investigations such as diagnostic laryngoscopy under general anesthesia or medical imaging, increasing the chance of finding more than one lesion. Recall was lowest for T1 hypopharyngeal tumors (20.6%), which represents a significant limitation of the current model. This limitation is particularly important because the primary clinical value of AI-assisted endoscopy is in detecting early-stage lesions that are often challenging to identify during routine examinations. The underrepresentation of stage 1 tumors in the dataset has likely contributed to this reduced performance (Table A1). Additionally, because this study used an object-level rather than a lesion-level analysis, it remains unclear whether intermittent malignant predictions would reliably detect these lesions throughout a complete endoscopic examination. This intermittent detection pattern is also suggested in the supplementary video materials. Previous studies indicate that successive algorithm updates can substantially improve diagnostic performance, suggesting that further optimization and expanded training datasets may enhance the detection of early-stage lesions.

The DL algorithm demonstrated strong discrimination between benign and (pre)malignant lesions, with an overall AUROC of 0.935. Within this malignancy-enriched internal test set, (pre)malignant lesions were correctly classified in 94.3% of cases, a clinically relevant rate in secondary referral settings where missed malignancies have substantial consequences. However, because of the composition of the test set, this estimate may not directly reflect performance in daily practice. Although the dataset was split at the patient level to prevent data leakage, the resulting test set contained 34.7% of all lesion frames and 50.5% of all non-lesion frames. This difference reflects variation in video length and lesion presence between patients rather than frame-level sampling. The higher proportion of non-lesion frames may have influenced the precision estimate, as precision is influenced by the number of FP detections. The result should be interpreted in the context of the underlying distribution of lesion types and subsites. This is particularly relevant because the prevalence of malignancy in the test set (65.3%) is higher than expected among lesions found in routine otorhinolaryngology clinics. In the prevalence sensitivity analysis (Table A3), the PPV decreased from 90.2% at the observed prevalence to 9.1% at a malignancy prevalence of 2%, whereas the NPV increased from 88.3% to 99.9%. Depending on the clinical setting, the predictive value of a malignant classification is strongly dependent on the underlying prevalence. In addition, the prevalence of malignancy may vary according to the location of the lesion in the UADT. Therefore, positive classifications would require cautious interpretation and clinical confirmation. At the same time, a high NPV may support the potential clinical utility of the algorithm as a tool to help rule out malignancy in a detected lesion in a low-prevalence setting. As these results are all on an object-level, and clinicians would aggregate predictions across all frames to reach a conclusion per lesion in a video, the clinical predictive values at the lesion level remain unknown and should be further investigated. Although excluding uncertain predictions (8% of detections) may have overestimated classification performance, retaining an uncertain class reflects the intended clinical application of the model. In the hypopharynx, specificity was highly imprecise and therefore not a reliable estimate (point estimate: 41.2%, 95% CI 0.0–1.000). This low estimate is unlikely to be explained by class imbalance in the test set, as specificity is prevalence-independent, but may instead reflect class imbalance in the training data, where malignant cases predominated and potentially biased model learning. However, given the extremely wide confidence interval, the data do not allow reliable conclusions regarding the model’s true hypopharyngeal specificity. Although the classification performance for vocal fold lesions was the lowest among the evaluated subsites despite the largest number of training samples, overall performance remained high. This result demonstrated the model’s robustness in this anatomically challenging region. The reduced performance is likely attributable to the greater visual heterogeneity of glottic lesions, which encompass a broad spectrum of benign entities that are difficult to distinguish from (pre)malignant lesions, including low-grade dysplasia and hyperkeratosis [15,16].

A model should be robust to variations in patient population, imaging conditions, and endoscopic acquisition settings before clinical deployment. According to a systematic review by Wilmes et al., most DL algorithms for UADT endoscopy demonstrate strong performance on static image datasets but lack validation in real-time endoscopic conditions, underscoring a critical translational gap [16,17,18,20]. The current study provides the first step towards addressing this gap by incorporating as many informative full-length endoscopy videos obtained during routine clinical practice as possible, while excluding videos or frames with insufficient visual quality for reliable analysis. The included videos retained common real-world challenges such as motion artifacts, variable illumination, and partial visual obstruction. While this supports robustness and generalizability, external validity cannot be established without independent validation in external centers with different case-mix and acquisition protocols.

To date, a study by Li et al. is the only comparable study reporting a real-time DL algorithm for recognition of laryngopharyngeal cancer [21]. This study primarily focuses on malignancy classification and does not elaborate on detection performance or benign lesion classification. Therefore, our study is the first to assess a real-time detection and classification model for benign and malignant laryngeal and hypopharyngeal lesions. Moreover, the present study provides T-classification and subsite-stratified performance analyses, which have not been previously reported. The QUAIDE and STARD-AI guidelines were followed to ensure transparent, structured reporting [24,25]. In addition, performance was evaluated at both the determined IoU threshold of 0.3 and the more strict threshold of 0.5 (Figure A1). This provides transparency regarding the effect of our IoU threshold on model performance. It was chosen to retain the threshold of 0.3 because the model is intended to function as an alerting tool rather than a precise delineation assist.

Several limitations should also be acknowledged. First, detection of early-stage tumors remains an important limitation. Recall for T1 tumors was 49.1% overall and only 20.6% for T1 hypopharyngeal carcinoma. Although these estimates were based on few lesion instances and therefore had considerable uncertainty, this result is particularly relevant because early-stage lesion detection is a primary aim of AI-assisted endoscopy. Second, because of the lower incidence of supraglottic and hypopharyngeal lesions, the corresponding datasets contained relatively few cases, particularly of benign lesions. While this reflects the inherent case distribution of these subsites in clinical practice, it limits the accuracy of classification performance estimates. Third, including the video frames increased the number of lesion instances in the database but reduced case diversity, as multiple similar frames of the same lesion were overrepresented. This may inflate performance estimates by reducing case-level variability and may decrease generalizability. Although the dataset included recordings from two endoscope systems, the model was only evaluated on an internal test set, not meeting the requirements for proper external validation. The absence of a clinical benchmark limits direct comparison with clinician performance, and prospective validation is needed to confirm the model’s performance in routine clinical practice. Furthermore, although the model demonstrated real-time-capable inference speed in retrospectively archived videos, real-time deployment in a prospective clinical setting remains untested. Another limitation is that frame-level bounding box annotations may not adequately capture the diffuse, irregular growth patterns characteristic of SCCs in the laryngopharyngeal tract. Segmentation models may be preferred in that case, potentially further improving model performance. The use of heterogeneous reference standards may additionally be considered a limitation. However, most lesions were histopathology confirmed, and videos were excluded from the database when persistent uncertainty regarding the nature of a lesion remained.

Addressing these limitations will require further multicenter data collection to increase lesion diversity and imaging heterogeneity. Extending the model to adjacent subsites, including the nasopharynx and oropharynx, may improve performance by learning from shared visual patterns across related anatomical regions. This represents a logical next step towards a fully integrated real-time laryngopharyngeal model. Future research should incorporate spatio-temporal data structures that maintain lesion identity across consecutive frames, thereby facilitating comprehensive lesion-level performance evaluation. Building on prior work from our research group in glottic lesions, further external validation and benchmarking against clinician performance are needed before the clinical value of the model can be established. Prospective studies comparing AI-assisted endoscopy with conventional, unassisted endoscopy are needed to evaluate whether AI assistance improves lesion detection and classification in real-time clinical practice.

## 5. Conclusions

The extended DL algorithm demonstrated strong detection performance and promising classification performance on glottic, supraglottic, and hypopharyngeal lesions during flexible endoscopy on an internal test set. Early-stage tumor detection remained challenging, which is an important limitation because early-stage lesion detection is a primary aim of AI-assisted endoscopy. These findings indicate the feasibility of a single real-time DL model for unified detection and classification of laryngeal and pharyngeal lesions, establishing a basis for further development. With additional validation, such a model could potentially support lesion detection and classification in outpatient clinics, including in anatomically complex regions. Nevertheless, future multicenter prospective studies with external validation and direct comparisons between AI-assisted and conventional endoscopy are necessary before clinical utility can be established.

## Figures and Tables

**Table 1:** Definitions of outcome measures for reporting detection and classification performance at the object level.

Metric	Definition
**Detection performance**	
IoU	The area of overlap of the predicted bounding box and the ground-truth bounding box, divided by the combined area of the predicted and ground-truth boxes.
TP	Correctly identified lesion, defined by an IoU of ≥0.3
FP	Predicted bounding box in absence of a true lesion
FN	Unidentified lesion, defined by no predicted bounding box or a bounding box with an IoU of <0.3
Precision	The ratio of true positive detections to all positive detections: $\frac{TP}{TP+FP}$
Recall	The ratio of actual lesions correctly detected by the algorithm: $\frac{TP}{TP+FN}$
AUPRC	Balance of precision and recall across all detection confidence thresholds
F1 score	Harmonic mean between precision and recall: $\frac{2\times precision\times recall}{precision+recall}$
**Classification performance**	
TP	A (pre)malignant lesion correctly classified as (pre)malignant
FP	A benign lesion incorrectly classified as (pre)malignant
TN	A benign lesion correctly classified as benign
FN	A (pre)malignant lesion incorrectly classified as benign
Sensitivity	The ratio of actual (pre)malignancies correctly classified as malignant by the algorithm: $\frac{TP}{TP+FN}$
Specificity	The ratio of actual benign lesions correctly classified as benign by the algorithm: $\frac{TN}{TN+FP}$
Coverage	The fraction of samples classified with sufficient confidence, defined as a classification confidence score below the lower threshold of 0.341 (benign) or above the upper threshold of 0.743 (malignant). Samples with an intermediate score are classified as uncertain.
AUROC	Summarizing discrimination between benign and (pre)malignant lesions across all classification thresholds, excluding the uncertain class. Note: the algorithm can still be confidently wrong within covered samples.
Accuracy	The proportion of all objects that were correctly classified, with uncertain predictions considered incorrect: $\frac{TP+TN}{TP+\;TN+FP+FN}$
Balanced accuracy	The average of sensitivity and specificity, giving equal weight to both classes: $\frac{Sensitivity+specificity}{2}$
PPV	The proportion of objects classified as malignant that were truly malignant: $\frac{TP}{TP+FP}$
NPV	The proportion of objects classified as benign that were truly benign: $\frac{TN}{TN+FN}$

Abbreviations: IoU, Intersection over union; FP, False positive; TP, True positive; FN, False negative; AUPRC, Area under the precision-recall curve (AUPRC); TN, True negative; AUROC, Area under the receiver operating curve; PPV, positive predictive value; NPV, negative predictive value.

**Table 2:** Diagnostic object-level detection performance of the DL algorithm on the test set, stratified by subsite and tumor T-classification.

	Metric Value (95% CI)	Unified	Subsite Glottic	Supraglottic	Hypopharynx
Performance on full test set					
Full set	Precision	0.921 (0.909–0.932)	0.905 (0.888–0.920)	0.932 (0.906–0.953)	0.961 (0.948–0.973)
	Recall	0.733 (0.698–0.766)	0.718 (0.674–0.763)	0.771 (0.704–0.833)	0.744 (0.674–0.808)
	AUPRC	0.868 (0.836–0.889)	0.846 (0.810–0.879)	0.884 (0.838–0.923)	0.891 (0.825–0.928)
	F1-score	0.816	0.801	0.844	0.839
Performance stratified by tumor classification stage					
T1	Precision	0.926 (0.828–0.967)	-	0.970 (0.933–0.985)	0.740 (0.517–0.829)
	Recall	0.491 (0.276–0.700)	-	0.655 (0.448–0.837)	0.206 (0.048–0.433)
	AUPRC	0.640 (0.388–0.834)	-	0.760 (0.517–0.913)	0.340 (0.134–0.650)
	F1-score	0.641	-	0.782	0.322
T2	Precision	0.948 (0.927–0.964)	-	0.930 (0.883–0.961)	0.959 (0.937–0.976)
	Recall	0.707 (0.624–0.790)	-	0.689 (0.554–0.826)	0.717 (0.615–0.814)
	AUPRC	0.839 (0.787–0.884)	-	0.841 (0.748–0.920)	0.837 (0.769–0.895)
	F1-score	0.810	-	0.790	0.823
T3	Precision	0.954 (0.934–0.969)	-	0.946 (0.915–0.968)	0.964 (0.943–0.981)
	Recall	0.851 (0.799–0.899)	-	0.877 (0.813–0.928)	0.819 (0.730–0.893)
	AUPRC	0.932 (0.901–0.958)	-	0.953 (0.919–0.978)	0.904 (0.857–0.948)
	F1-score	0.899	-	0.910	0.884
T4	Precision	0.938 (0.879–0.979)	-	0.863 (0.677–0.962)	0.978 (0.957–0.992)
	Recall	0.800 (0.694–0.874)	-	0.781 (0.541–0.910)	0.809 (0.682–0.890)
	AUPRC	0.917 (0.842–0.956)	-	0.854 (0.581–0.960)	0.938 (0.874–0.968)
	F1-score	0.863	-	0.820	0.887

Abbreviations: AUPRC, Area under the precision-recall curve; CI, Confidence interval. T-stage specific glottic results were not available for stratified analysis.

**Table 3:** Binary classification performance of the DL algorithm on the internal test set on an object-level, stratified by subsite.

Metric Value (95% CI)	Unified	Glottic	Subsite Supraglottic	Hypopharynx
Sensitivity	0.943 (0.918–0.965)	0.880 (0.825–0.924)	0.992 (0.984–0.997)	0.999 (0.998-1.000) *
Specificity	0.807 (0.716–0.887)	0.806 (0.700–0.890)	0.874 (0.774–0.956)	0.412 (0.000–1.000) **
AUROC	0.935 (0.899–0.962)	0.881 (0.816–0.930)	0.991 (0.982–0.997)	0.980 (0.756–1.000) **
Coverage	0.920 (0.906–0.934)	0.879 (0.856–0.899)	0.976 (0.961–0.988)	0.995 (0.991–0.998)
Accuracy	0.836	0.745	0.962	0.994 ***
Balanced accuracy	0.875	0.843	0.933	0.706

Abbreviations: AUROC, Area under the receiver operating curve; CI, Confidence Interval. * The hypopharynx dataset is highly imbalanced (99.5% malignant), which reflects daily clinical practice, however, results should be interpreted accordingly. ** Hypopharyngeal specificity and AUROC estimates are derived from only 17 confidently classified benign instances and should therefore be interpreted with caution. *** As accuracy may be overestimated due to the imbalance in the dataset, the balanced accuracy could provide a more informative measure of the DL algorithm performance.

## Data Availability

Data are available upon reasonable request and subject to institutional ethics committee approval.

## Supplementary Materials

The following supporting information can be downloaded at: https://doi.org/10.5281/zenodo.20996838, S1: technical description of the Zeno AI model; Video S1: full-length endoscopic video of the Zeno AI algorithm, the model detects and classifies a T1 hypopharyngeal carcinoma in the right pyriform sinus with a weak detection performance; Video S2: full-length endoscopic video of the Zeno AI algorithm, the model detects and classifies a T1 hypopharyngeal carcinoma in the left pyriform sinus with a moderately strong detection performance.

## Abbreviations

The following abbreviations are used in this manuscript:

HNC	Head and neck cancer
SCC	Squamous cell carcinoma
UADT	Upper aerodigestive tract
HD	High-definition
WLI	White-light imaging
NBI	Narrow-band imaging
AI	Artificial Intelligence
DL	Deep learning
Radboudumc	Radboud University Medical Center
UMCG	University Medical Center Groningen
MH	Martini Hospital
CVAT	Computer Vision Annotation Tool
YOLO	You Only Look Once
STARD-AI	Standards for Reporting Diagnostic Accuracy using Artificial Intelligence
QUAIDE	Quality Assessment of Preclinical AI Studies in Diagnostic Endoscopy
AUPRC	Area under the precision-recall curve
AUROC	Area under the receiver operating characteristic curve
IoU	Intersection-over-union
CI	Confidence Interval
TP	True positive
TN	True negative
FN	False negative
PPV	Positive predictive value
NPV	Negative predictive value

## Appendix A

**Table A1:** Specifications of supraglottic and hypopharyngeal benign and malignant cases added to the Zeno AI database. Numbers represent lesion instance counts.

Lesion Type	Supraglottic Instances (n)	Hypopharynx Instances (n)
T1 carcinoma	1338	1169
T2 carcinoma	5363	8650
T3 carcinoma	8500	6584
T4 carcinoma	2297	4803
Low-grade dysplasia	42	0
Papilloma	74	0
Polyp	97	17
Hyperkeratosis	497	143
Cyst	1793	657
Reinke’s edema	440	0

**Table A2:** Frequency and proportion of videos and specific lesion entities in the total Zeno AI database and in the test set.

Lesion Type	Total Database (n, %)	Test Set (n, %)
Videos with lesion	1336	400
Glottic	846 (63.3)	255 (63.8)
Supraglottic	213 (15.9)	62 (15.5)
Hypopharynx	277 (20.7)	83 (20.7)
Lesion frames	96,095 (67.6)	33,325 (58.9)
Videos without lesion	123	33
Non-lesion frames	46,061 (32.4)	23,297 (41.1)
Number of instances		
(Pre)malignancy	70,570 (63.9)	24,818 (65.3)
Papilloma	12,615 (11.4)	4508 (11.9)
Polyp	6588 (6.0)	2367 (6.2)
Cyst	6790 (6.2)	2241 (5.9)
Hyperkeratosis	5300 (4.8)	1529 (4.0)
Reinke’s edema	3479 (3.1)	1168 (3.1)
Low-grade dysplasia	1510 (1.4)	216 (0.6)
Hyperplasia	1758 (1.6)	520 (1.4)
Granuloma	1423 (1.3)	588 (1.6)
Inflammation	243 (0.2)	10 (0.0)
Nodule	114 (0.1)	0 (0.0)

**Table A3:** Sensitivity analysis of positive and negative predictive values across different malignancy prevalence scenarios. Abbreviations: PPV, positive predictive value; NPV, negative predictive value.

Scenario	Malignancy Prevalence	Sensitivity	Specificity	PPV	NPV
Study prevalence	0.653	0.943	0.807	0.902	0.883
33% malignant	0.33	0.943	0.807	0.707	0.966
10% malignant	0.1	0.943	0.807	0.352	0.992
2% malignant	0.02	0.943	0.807	0.091	0.999
